# Pulmonary function in a cohort of heart-healthy individuals from Northern Sweden—a comparison with discordant reference values

**DOI:** 10.1186/s12890-023-02403-w

**Published:** 2023-04-05

**Authors:** Sofia Erelund, Kjell Karp, Sandra Arvidsson, Bengt Johansson, Nina Sundström, Urban Wiklund

**Affiliations:** 1 Department of Surgery and Perioperative Sciences, Clinical Physiology, Umeå University, 901 87 Umeå, Sweden; 2 Department of Radiation Sciences, Radiation Physics, Biomedical Engineering, Umeå University, Umeå, Sweden

**Keywords:** Spirometry, Lung function, Clinical physiology, Reference values, Linear regression

## Abstract

**Background:**

Dynamic spirometry is an important investigation to differentiate between impaired and normal lung function. This study aimed to evaluate the results of lung function testing in a cohort of subjects from Northern Sweden without any known heart or pulmonary disease. Our focus was to compare with two reference materials that have showed differences in the age-dependency of lung function in Swedish subjects.

**Methods:**

The study population consisted of 285 healthy adults (148 males, 52%) between 20–90 years of age. The subjects had been randomly selected from the population register for inclusion in a study investigating cardiac function in heart-healthy subjects, but were also assessed with dynamic spirometry. At least seven percent reported smoking. Sixteen subjects presented with pulmonary functional impairments and were excluded from the current study. The sex-specific age-dependency in lung volumes was estimated using the LMS model, where non-linear equations were derived for the mean value (M), the location (L) or skewness, and the scatter (S) or coefficient of variation. This model of the observed lung function data was compared with reference values given by the original LMS model published by the Global Lung Initiative (GLI), and with the model from the recent Obstructive Lung Disease In Norrbotten (OLIN) study, where higher reference values were presented for Swedish subjects than those given by the GLI model.

**Results:**

No differences were found in the age-dependency of pulmonary function between the LMS model developed in the study and the OLIN model. Although the study group included smokers, the original GLI reference values suggested significantly lower normal values of FEV_1_ (forced expiratory volume) and FVC (forced vital capacity), and consequently fewer subjects below the lower limit of normality, than both the rederived LMS and OLIN models.

**Conclusions:**

Our results are in line with previous reports and support that the original GLI reference values underestimate pulmonary function in the adult Swedish population. This underestimation could be reduced by updating the coefficients in the underlying LMS model based on a larger cohort of Swedish citizens than was available in this study.

**Supplementary Information:**

The online version contains supplementary material available at 10.1186/s12890-023-02403-w.

## Background

Dynamic spirometry measures the ventilatory function of the lungs and airways. There are a number of indicators for spirometry testing, such as evaluation of respiratory symptoms, preoperative risk assessment, health monitoring, monitoring of lung disease, evaluating medical treatment and screening and monitoring individuals exposed to harmful substances [1, 2]. Spirometry data is thus used to determine if lung function is impaired in an individual and to determine whether the aetiology is obstructive, restrictive or a combination of both pathophysiologies. Ageing is an important contributing factor to reduction of the lung function [3], irrespective of illness [4]. Other factors which affect lung function include body size, in particular height, and smoking history.

Interpretation of lung function test data must always be performed in conjunction with relevant and validated reference material for the investigated population [5]. Over the past decades, numerous articles have been published in this field making the selection of appropriate reference materials more complex [6]. The European Respiratory Society (ERS) and American Thoracic Society (ATS) recommended the reference values that were published by the Global Lung Function Initiative (GLI) and which were updated for various ethnic groups in 2012 [7]. The GLI reference values were determined based on material gathered from 57,395 subjects from South America, North Africa, USA, Australia, Israel and Sweden. The proportion of the study population consisting of Nordic subjects was low and studies have since indicated that the GLI reference values are suboptimal for evaluating the lung function in Caucasian subgroups in Sweden and Finland [8–10], for example; the GLI reference values were found to be lower than observed lung volumes in Swedish females [8].

This study focused on the spirometry testing in a cohort of 285 subjects with normal heart function from the general population of Northern Sweden. Cardiac data from this cohort in the form of echocardiographic and electrocardiographic (ECG) examinations have been published previously [11, 12], however this article is the first to present information gathered from the dynamic spirometry testing on this cohort. The present study aimed to evaluate the observed lung function in this cohort based on: a) the present GLI reference values, and b) the reference values suggested in the Obstructive Lung Disease In Norrbotten (OLIN) study [13] which is based on subjects from Northern Sweden. To compare how the observed lung function in this cohort differed from the OLIN and original GLI models, we applied the same mathematical model as used in the GLI study.

## Methods

The study group that was used for evaluation of different lung function models were originally included in a population-based study of cardiac function performed in Umeå, Northern Sweden during the years 1998–2000 [11, 12]. 1000 subjects (50% females) were randomized from the Swedish Tax Agency's register, with year of birth ranging from 1910 to 1977. These subjects received a letter regarding the study's content and an invition to participate. The inclusion criteria were; absence of any known lung/airway disease, absence of any cardiovascular and/or systemic disease, and no use of medications that could be expected to affect heart and/or lung function. The overall health status was determined during a telephone interview. The subjects remaining after this interview reported further details regarding their health status in a questionnaire before final inclusion. Subjects with hypertension, diabetes, hyperlipidaemia, previous stroke, previous transient ischemic attack, rheumatic fever and/or intermittent claudication were excluded. The final study cohort consisted of 285 subjects, evenly distributed between 20–90 years of age. The smoking habits of the subjects were examined based on a questionnaire, however the question regarding smoking was only added for the last 146 subjects of the study population. Of those, forteen subjects (10%, two males and twelve females) reported current smoking, and seven subjects (5%, all males) were former smokers.

All were investigated with: a) echocardiography, where all presented with normal findings [12]; b) electrocardiography (ECG), where 83 had minor pathological findings [11]; and c) dynamic spirometry. Testing and analyses was performed by experienced medical technicians and physicians. The study was approved by the Regional Ethics Review Board in Umeå, Sweden (Dnr 98–129), and was performed in accordance with the Declaration of Helsinki. All subjects gave their informed written consent to participate.

### Spirometry testing

Dynamic spirometry was measured by flow and volume during forced exhalation. The results were presented as a flow-volume graph (F/V-graph) and as numeric variables. This study assessed forced vital capacity (FVC), forced expiratory volume for one second (FEV_1)_ and FEV_1_/FVC ratio. Jaeger MasterLab Transfer spirometer (Erich Jaeger GmbH, Würzburg, Germany) was used for spirometry testing.

The equipment was calibrated each morning and the procedure followed the ATS/ERS recommendations [14]. The examination began with three calm tidal breaths, followed by a maximum inhalation before a maximum forced exhalation. The duration of exhalation was at least six seconds. Measurements were repeated at least three times with a short rest period between each test, and with a duplicability criteria of ≤ 5% variation from the second highest value. The highest recorded FEV_1_ value was recorded. Maximum value of the FVC was used when the FEV_1_ / FVC ratio was calculated.

### Clinical evaluation

Subjects with pathological findings were identified based on a clinical evaluation of reports from the spirometry examination. The reports were reviewed by one investigator (SE). The normal values were defined according to the ATS/ERS guidelines [1], where the lower limit of normality (LLN) corresponds to the 5th percentile of a normal population. The definition of airway obstruction was FEV_1_/FVC < LLN, and the definition of small FVC was FVC < LLN [15]. Those who had abnormal values were reviewed by a physican with expertice in lung function testing; they were also excluded from further analyses. The collection of data and the interpretation were not contemporary, therefore the tests were carried out according to the 1994 ATS/ERS guidelines [14] and the interpretation according to the updated version in 2019 [1].

### Model of the age-dependency in spirometry data

Many different models of how spirometric variables change with age have been suggested. To compare the age-dependency of the spirometry data in this current study with previously published reference values we used the same mathematical model used to determine the GLI reference values [7]. The LMS model is based on logarithmically transformed data and splines, where the fluctuations of data over different ages is described using three characteristics: the location (L), the mean value (M) and the scatter (S). The S corresponds to fluctuations around the M and is estimated by the coefficient of variation (CV), which in turn is given by the ratio between the M and standard deviation (SD). The L is an index of the skewness of the data around the M, but we assumed that this did not deviate from a normal distribution, which corresponds to L = 1. This assumption was verified by analyzing model errors as described below. The corresponding models for the age-related changes in the M and fluctuations around the mean value are given by:

$$M\left(age,height\right)={\mathrm{exp}(B}_{0}+{B}_{1}*\mathrm{log}(height)+{B}_{2}*\mathrm{log}(age)+Mspline)$$

$$S\left(age\right)={\mathrm{exp}(B}_{0}+{B}_{1}*\mathrm{log}(age)+Sspline)$$

The spline can be regarded as a more complex model of the interaction between age and height than the product of these two factors, which often is included in linear regression models. In order to compare with other reference equations, the coefficients and splines in the new LMS model were determined for FEV_1_, FVC, and FEV_1_/FVC with different models for males and females. This rederived LMS model is hence forth referred to as the rLMS model.

To examine the sampling variability of the estimated model, data were randomly partioned into ten subsets of equal size. Ten different LMS models were estimated based on nine subsets, where each subset was excluded in one model. The range of these ten models at different ages was then determined and presented graphically.

### Comparison with other spirometry data models

The new LMS models based on the observed data in our study group was compared with the GLI reference values [7], where the published coefficients and splines in the LMS model for Caucasians were used. The GLI reference values cover almost the entire lifespan, ranging from 3–95 years of age.

We also compared our results with the predicted values based on the OLIN model [13] which is based on a sample from 501 healthy, non-smoking adults (244 females, 257 males), aged 22–91 years, from northern Sweden. In this model the age-dependency of the spirometry variables is estimated by polynomial splines with different shape in four different age ranges. These four splines are merged so that the resulting curve is smooth over the entire age range.

### Statistical analysis

For clinical characteristics and spirometry variables, means and standard deviations were calculated. Linear regression was used to assess the association between age and height. The evaluation of different models of spirometry data based on age-dependency, was performed by predicting the mean and LLN at different ages. For each subject the corresponding predicted values and residuals were calculated based on the subject’s age, height and gender for all included models. The normality of the residuals, i.e., the difference between the measured values and the predicted values, were tested with the Kolmogorov–Smirnov test, and the deviation of the mean from zero was tested with the one-sample *t*-test.

Differences in the distribution of residuals were tested with the two-samples Kolmogorov–Smirnov test and with analysis of variance (ANOVA). Data were also converted to Z-scores to determine how much a measured value deviated from the predicted value. This was performed by subtracting the predicted mean and then dividing by the corresponding standard deviation based on the subject’s age, height and gender. The distribution of Z-scores were compared for the different models using density plots. LLN corresponds to the Z-score $-1.64$. Subjects with Z-scores below the LLN based on the reference values given by each model were identified. The null hypothesis was rejected for *p*-values < 0.05.

Data processing and statistical analyses were performed in Microsoft Office Excel 2016 (Microsoft corporation, Redmond, WA, USA) and Matlab R2022a (Mathworks Inc, Natick, MA, USA). All computational analyses were performed using R version 4.1.3 [16] and the GAMLSS package version 5.4–1 (https://cran.r-project.org/package=gamlss)).

## Results

### Spirometry

Anthropometric and spirometry data for the 285 subjects who participated in spirometry testing is displayed in Tables 1 and 2. The mean Body mass index (BMI) for females was 24.2 and 25.2 for males. In females, the annual decrease in height was 0.19 cm/year (CI 0.13–0.24, r^2^ = 0.27, F = 48.8, *p* < 0.001), whereas the annual decrease in males was 0.14 cm/year (CI 0.08–0.19, r^2^ = 0.15, F = 26.0, *p* < 0.001).

**Table 1:** Clinical characteristics of female participants

**Females**	< 40 years (*N* = 27)		40–60 years (*N* = 55)		> 60 years (*N* = 55)	
	Mean (SD)	Range	Mean (SD)	Range	Mean (SD)	Range
Weight (kg)	64.4 (11.0)	44–92	66.1 (10.2)	48–93	63.9 (7.6)	45–78
Height (cm)	167.5 (5.9)	157–181	165.8 (5.7)	151–183	159.7 (6.1)	146–174
BMI (kg/m^2^)	22.9 (3.6)	17.4–32.3	24.0 (3.3)	18.4–32.6	25.1 (3.2)	18.7–33.8
FVC (l)	4.09 (0.49)	3.34–5.06	3.90 (0.50)	2.78–5.17	2.76 (0.58)	1.30–4.02
FEV_1_(l)	3.40 (0.48)	2.7–4.15	3.09 (0.40)	2.26–4.03	2.10 (0.51)	0.90–3.22
FEV_1_/FVC	0.83 (0.06)	0.65–0.92	0.79 (0.06)	0.64–0.95	0.75 (0.07)	0.52–0.95

*BMI* Body mass index

*FVC* Forced expiratory vital capacity

*FEV*_*1*_ Forced expiratory volume in one second

**Table 2:** Clinical characteristics of male participants

**Males**	< 40 years (*N* = 31)		40–60 years (*N* = 59)		> 60 years (*N* = 58)	
	Mean (SD)	Range	Mean (SD)	Range	Mean (SD)	Range
Weight (kg)	79.5 (12.9)	60–106	84.5 (13.5)	59–124	76.7 (12.2)	57–105
Height (cm)	189.6 (7.4)	167–196	179.8 (5.3)	168–191	175.6 (6.3)	157–189
BMI (kg/m^2^)	24.3 (3.4)	19.6–33.1	26.1 (3.72)	18.8–35.1	24.8 (3.3)	18.6–32.6
FVC (l)	5.65 (0.83)	4.31–7.26	5.35 (0.77)	3.75–6.69	4.29 (0.66)	2.85–5.80
FEV_1_(l)	4.55 (0.64)	3.17–5.93	4.10 (0.74)	1.91–5.26	3.10 (0.59)	1.37–4.68
FEV_1_/FVC	0.81 (0.06)	0.65–0.97	0.76 (0.07)	0.51–0.88	0.72 (0.08)	0.43–0.87

*BMI* Body mass index

*FVC* Forced expiratory vital capacity

*FEV*_*1*_ Forced expiratory volume in one second

The clinical evaluation of spirometry reports showed that sixteen subjects (6%, seven females and nine males) presented with FEV_1_/FVC < LLN, where two female subjects also presented with small FVC. In addition, one of the male subjects presented with FEV_1_/FVC = 0.43, i.e., findings indicating Chronic obstructive pulmonary disease (COPD). These sixteen subjects with pathological findings where thus excluded from the study.

**Fig. 1:**
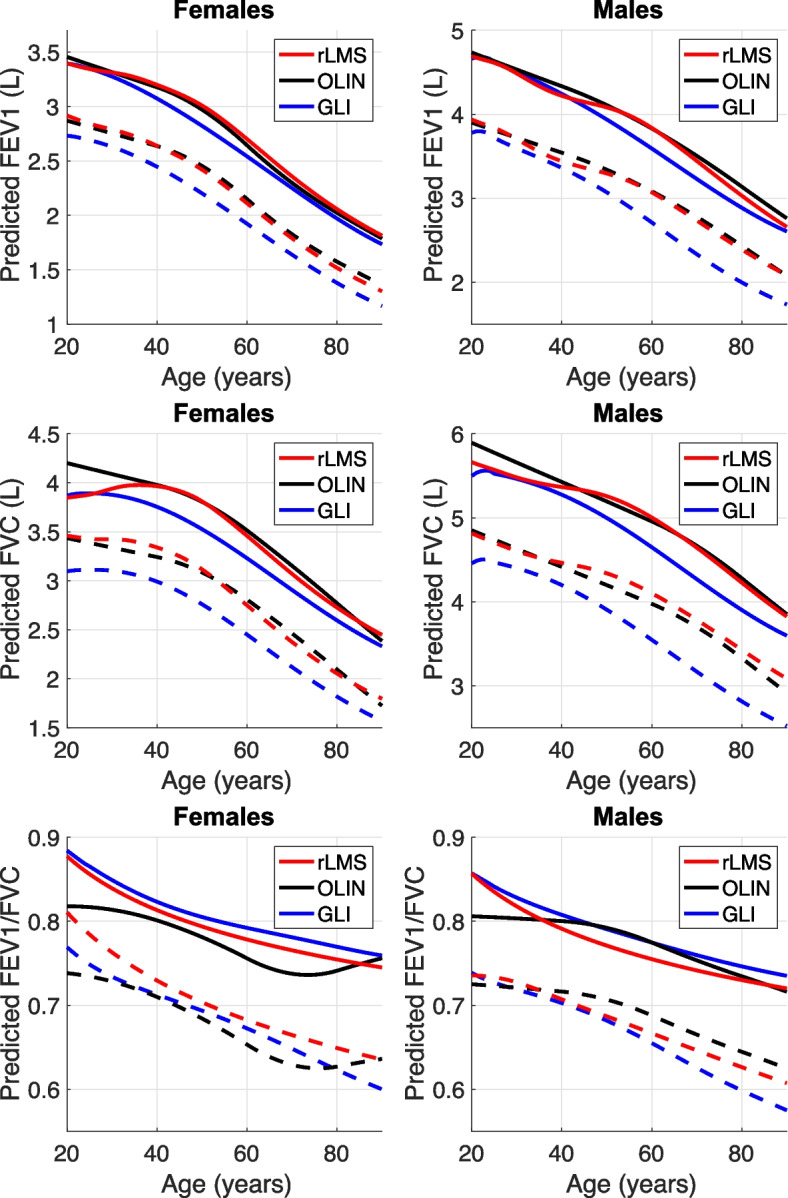
Comparison between our (rLMS) and the two other models of sex and age dependent changes in FEV_1._, FVC and FEV_1._/FVC. Predicted reference values of mean (solid lines) and LLN (dotted lines) are plotted against age for a female with height 164 cm and a male with hight 178 cm

**Fig. 2:**
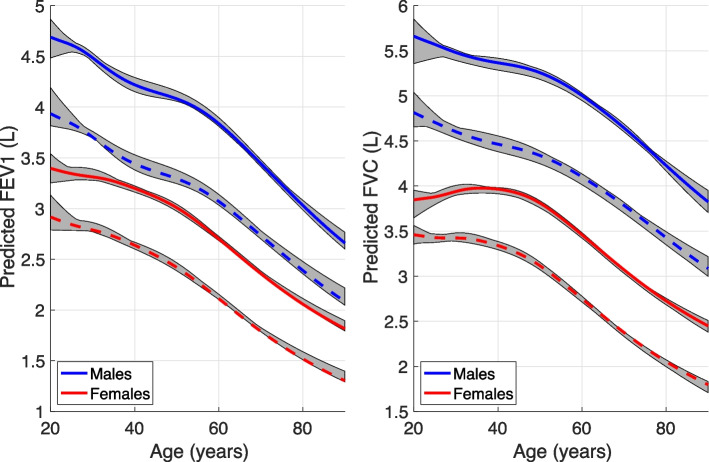
Sampling variability of the rLMS model of FEV_1_ and FVC. Solid and dashed lines show the mean and LLN for the models based on all female and male subjects, and the gray area the range of the differences based on 10 different subsets

The rLMS model was estimated based on the remaining 269 subjects with normal spirometry results. Figure 1 shows a comparison between the predicted values based on the rLMS, GLI and OLIN models for FEV_1_, FVC and FEV_1_/FVC, respectively. The age dependent changes in mean and LLN are shown for males of height 178 cm and females of height 154 cm, which were the sex-specific average heights in our study group. The rLMS models are presented in the look-up table in the Supplementary material. The variability of the estimated rLMS model is shown in Fig. 2. The range of the ten models for different subsets showed that the variability was relatively small, except at young ages.

Table 3 shows a comparison between the residuals from all three models. The Kolmogorov–Smirnov test showed that the distribution of the residuals from all three models did not deviate from a normal distribution (data not shown). The mean of the residuals was only significantly different from zero for the GLI based models of FEV_1_, FVC and FEV_1_/FVC. Morover, the ANOVA showed that GLI gave higher mean values of residuals, or eqivalently lower predicted, FEV_1_ and FVC than both the rLMS and OLIN models, whereas no differences were found between the rLMS and OLIN models.

**Table 3:** Residuals based on the different models

	rLMS Mean (SD)	OLIN Mean (SD)	GLI Mean (SD)	ANOVA *p*-value	rLMS vs OLIN *p*-value	rLMS vs GLI *p*-value	GLI vs OLIN *p*-value
FEV_1_	0.000 (0.405)	-0.002 (0.413)	0.106* (0.419)	0.003	0.95	0.003	0.003
FVC	0.000 (0.469)	-0.029 (0.481)	0.197* (0.487)	< 0.001	0.48	< 0.001	< 0.001
FEV_1_/FVC	0.000 (0.057)	0.005 (0.059)	-0.015* (0.057)	< 0.001	0.25	0.003	< 0.001

Residuals are calculated as observed-predicted value. p-values are derived from ANOVA and post-hoc paired t-tests

*FVC* Forced expiratory vital capacity

*FEV*_*1*_ Forced expiratory volume in one second

^*^ p < 0.05 one-sample t-test of mean

**Fig. 3:**
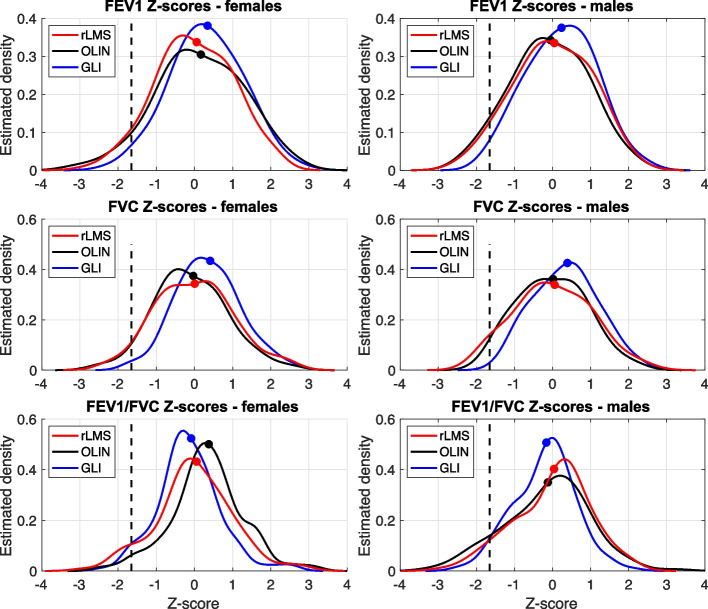
Distribution of Z-scores based on the rLMS, OLIN and GLI reference equations for FEV_1_, FVC and FEV_1_/FVC in females and males. Dots show mean value, and dashed lines show LLN

Figure 3 shows the overall distribution of Z-scores based on the predicted values for the 269 included study subjects. In general, the rLMS and OLIN resulted in similar distributions of Z-scores, whereas the GLI model showed both a higher mean and a lower density in the region with Z-scores below LLN for FEV_1_ and FVC than both the rLMS and OLIN model. Table 4 presents the number of subjects with spirometric values below the LLN.

**Table 4:** Proportion of the included 131 females and 138 males that presented with Z-scores < LLN

< LLN	OLIN		GLI		rLMS	
	Females	Males	Females	Males	Females	Males
FEV_1_	6.9%	7.2%	1.5%	1.4%	5.3%	5.8%
FVC	3.8%	2.2%	0.8%	0.0%	3.8%	5.1%
FEV_1_/FVC	2.3%	11.6%	3.8%	3.6%	6.9%	6.5%

*FVC* Forced expiratory vital capacity

*FEV*_*1*_ Forced expiratory volume in one second

## Discussion

This study evaluated the lung function in a cohort of persons without cardiac disease from northern Sweden, with a particular focus on comparing with the discordant GLI and OLIN reference values. We found that the re-derived LMS model of the observed lung function in this cohort, was comparable to the OLIN model which is based on subjects from the same region [10, 13]. In addition, since the GLI reference values based on an international population were lower than the measured values in this cohort, we were able to confirm previous findings that the GLI reference values are suboptimal for the population of Sweden [8, 9]. Based on the initial clinical evaluation, sixteen subjects (6%) were excluded due to pathological lung function test results. As expected from the definintion of LLN, other subjects also presented with at least one lung function parameter below LLN, however, fewer subjects were classified as having reduced lung function based on the GLI reference values as compared to the OLIN model.

Reference equations should be validated for each population as each population will have different characteristics [5, 17, 18]. The objective when producing the GLI model was to create specific spirometry reference values for different parts of the world. The GLI model is based on data from different centers across Europe. However, there are differences in socio-economic, environment, nutrition and ethnicity features between the Nordic countries and other parts of Europe [8]. Several studies have shown that there is a need to develop separate reference equations for the Nordic countries. Backman et al. concluded in their study that the use of reference values from GLI may lead to incorrect interpretations regarding airway obstruction in the Swedish population, especially in females [8]. Brisman et al. also highlighted that the range of the GLI reference values is too wide in Swedish adults [9]. The GLI reference values also underestimate lung volumes in the Finnish population, as revealed by a study based on 1000 healthy non-smokers between 18–83 years of age [10]. On the contrary, the GLI reference values appear to agree with Norwegian data and consequently were recommended as reference values in the Norwegian healthcare system [19].

When comparing the rLMS model with the other models, the predicted mean FEV_1_ and FVC values corresponded well with the OLIN model in particular within the age range of 40–90 years, where the GLI model gave lower predicted mean than the other two models. However, for both FEV_1_ and FVC the GLI model gave markedly lower LLN at all ages than both rLMS and OLIN. The grading of respiratory disease frequently relies on FEV_1_, thus using the GLI model reference data may lead to bias in lung disease classification in Swedish citizens [8]. Thus, both our study and previous reports illustrate that it is important to have adequate and accurate reference values reflecting the anthropomorphic and demographic characteristics of the population being assessed to ensure accurate lung function test results and diagnoses.

This study has shown that it is possible to use the same mathematical model as in the GLI to derive specific reference values also for the Swedish population, but these reference values should probably be based on a larger cohort than was available in our study [18]. In particular since all models are dependent on the data that was used to estimate the parameters in the model. Therefore differences in the shape of the estimated age and height dependency of models from different studies can be expected [18]. This is illustrated by the deviations between our model and OLIN at some ages, but also by the relatively low sampling variability between LMS models based on different subsets of our data.

Lung function models based on linear and quadratic regression have the limitation that they predict lung volumes that decrease towards zero at very old age[20–22]. Both the LMS and OLIN models have introduced more complex non-linear models that provide a more reliable result of gender-specific spirometry outcomes, while also accounting for the subject’s age and height. Height has an impact on the lung function, as the vital capacity (VC) is affected by height [23]. With increasing age it is known that height decreases, in our study we found an annual decrease of 0.19 cm/year in females and 0.14 cm/year in males. In a 2018 report from Statistics Sweden subjects aged 20 years were found to be 8 cm taller than subjects aged 85 years (available at https://www.scb.se/hitta-statistik/sok/?query=Hälsa-2018&lang=sv). Tall, elderly people are still rather uncommon in many countries, consequently making it hard to create accurate reference values for tall subjects of old age. However, tall people of old age will probably become more and more common in the Nordic countries, where the adult height has increased progressively in the last decades [24].. This finding was also described by Kainu et al. who found that the predicted FEV_1_/FVC ratio in females and males was lower than seen in the GLI model, and that the effect of height on predicted FEV_1_/FVC was larger than in the GLI model [10].

In the present study, sixteen of the ‘heart healthy subjects’ presented with signs of reduced lung function, where one subject presented signs of undiagnosed COPD. It is established by previous studies that, with normal aging, there is a continual decline of dynamic lung volumes [25]. Both FVC and FEV_1_ decline with age, and the F/V-curve can present as a sign of, or mimic COPD in elderly subjects [3, 26]. Due to age-related loss of elastic tissue in the lung parenchyma, the lungs are increasingly prone to false obstruction when there is a significant fall in pressure during expiration. The difference between this ‘false obstruction’ and a true obstruction may be difficult to detect based on FEV_1_ alone [27]. Age also increases the closing volume and the residual volume of the lungs, and furthermore the alveolar wall structure breaks down. Moreover, the prevalence of dyspnoea has been established to increase with age [28]. This physiological decline in lung function therefore make it very difficult to determine robust values of lower normal limits, especially at old age [25, 27].

To compare all models based on the same data, we also determined Z-scores based on the reference values given by the GLI and OLIN models. According to the definition of LLN, approximately 14 subjects (5%) should present with lung function values below LLN also in a normal population. Nineteen subjects with normal spirometry findings presented with Z-scores < LLN for FEV_1_ based on the OLIN reference values, wheras only four subjects were classified as pathological according the GLI reference values. The somewhat high number of subjects with low Z-scores based on the OLIN model could be due to the fact that our study included a number of smokers. However, despite this we did not find any significant differences between the predicted means with our rLMS model and the OLIN model, as is shown when comparing residuals, the estimated age-dependency and the distribution of Z-scores.

A decline in FEV_1_ in smokers has been reported before, e.g. in the clinical trial by Anthonisen et al. where males who smoked during the study time (11y) had a decline by 66 ml/ year and females declined by 54 ml/year [29]. Considering that at least 7% of subjects in our cohort were smokers, or former smokers, it is feasible to assume that our new model should have predicted lower mean lung volumes in our study group than given by the OLIN model but this was not the case. Even though we also had other potential smokers/ex-smokers in the study group, our model also predicted higher mean lung volumes than those given by the GLI model in this inhomogenous cohort. This was also the case in the study by Kainu et al. who had former smokers in their study [10]. This further supports the hypothesis that OLIN is a more relevant model than GLI's current reference values for the Swedish and the Finnish population. Nevertheless, a limitation of our study is that it is a relatively small study and that the selection criteria mainly focused on recruiting subjects that were suitable for inclusion in a normal material in studies of cardiac function. Another weakness is that smoking status was not investigated in the first round of inclusion. In addition, it was unclear who was a smoker and who was a former smoker. Still, this study provides a cross-sectional picture of lung function in a population, given that smokers are found in all populations.

In conclusion, our results support that the original GLI reference values are suboptimal for evaluating pulmonary function in the Swedish population. Despite the limitations of our material, we were also able to derive a LMS model of the observed lung function in this cohort that was equivalent to the OLIN model. The advantage with the GLI model is that it can be used for several etnical groups. Thus, the major contribution of this study was the finding that the difference between the reference values given by the OLIN and GLI models could be reduced by updating the coefficients in the underlying LMS model. However, to ensure optimal accuracy we recommend that the new LMS model of lung function in Swedish citizens is based on a larger cohort of subjects than was available in this study.

## Supplementary Information

**Additional file 1.** rLMS Lookup Table

## Data Availability

The datasets used and/or analysed during the current study are available from the corresponding author on reasonable request.

## Abbreviations

ATS: American thoracic society
BMI: Body mass index
COPD: Chronic obstructive pulmonary disease
ECG: Electrocardiography
ERS: European respiratory society
FEV_1_: Forced expiratory volume for one second
FVC: Forced vital capacity
GLI: Global lung function initiative
LLN: Lower limit of normality
OLIN: Obstructive lung function in Northern Sweden
PEF: Peak expiratory flow
rLMS: Acronym for the rederived LMS model
VC: Vital capacity
